# Inhibition of DNMT1 and ERRα crosstalk suppresses breast cancer via derepression of *IRF4*

**DOI:** 10.1038/s41388-020-01438-1

**Published:** 2020-08-27

**Authors:** Mathieu Vernier, Shawn McGuirk, Catherine R. Dufour, Liangxinyi Wan, Etienne Audet-Walsh, Julie St-Pierre, Vincent Giguère

**Affiliations:** 1 Goodman Cancer Research Centre, McGill University, Montréal, H3A 1A3 QC Canada; 2 Departments of Biochemistry, Medicine and Oncology, Faculty of Medicine, McGill University, Montréal, H3G 1Y6 QC Canada; 3 Present Address: Département de Médecine Moléculaire, Faculté de Médicine, Centre de Recherche du CHU de Québec, Université Laval, Québec, QC G1V 4G2 Canada; 4 Present Address: Department of Biochemistry, Microbiology and Immunology, Ottawa Institute of Systems Biology, Faculty of Medicine, University of Ottawa, Ottawa, ON K1H 8M5 Canada

**Keywords:** Breast cancer, DNA methylation

## Abstract

DNA methylation is implicated in the acquisition of malignant phenotypes, and the use of epigenetic modulating drugs is a promising anti-cancer therapeutic strategy. 5-aza-2’deoxycytidine (decitabine, 5-azadC) is an FDA-approved DNA methyltransferase (DNMT) inhibitor with proven effectiveness against hematological malignancies and more recently triple-negative breast cancer (BC). Herein, genetic or pharmacological studies uncovered a hitherto unknown feedforward molecular link between DNMT1 and the estrogen related receptor α (ERRα), a key transcriptional regulator of cellular metabolism. Mechanistically, DNMT1 promotes ERRα stability which in turn couples DNMT1 transcription with that of the methionine cycle and S-adenosylmethionine synthesis to drive DNA methylation. In vitro and in vivo investigation using a pre-clinical mouse model of BC demonstrated a clear therapeutic advantage for combined administration of the ERRα inhibitor C29 with 5-azadC. A large-scale bisulfite genomic sequencing analysis revealed specific methylation perturbations fostering the discovery that reversal of promoter hypermethylation and consequently derepression of the tumor suppressor gene, *IRF4*, is a factor underlying the observed BC suppressive effects. This work thus uncovers a critical role of ERRα in the crosstalk between transcriptional control of metabolism and epigenetics and illustrates the potential for targeting ERRα in combination with DNMT inhibitors for BC treatment and other epigenetics-driven malignancies.

## Introduction

Methylation of DNA is an evolutionarily conserved mechanism that allows control of gene expression by modulating chromatin accessibility to specific transcription factors (TFs) [1, 2]. This epigenetic process is crucial for proper mammalian development, essential for cellular differentiation and plays a determinant role in maintaining genomic stability [3–5]. Aberrant DNA methylation patterns have been observed in a large variety of diseases including obesity, diabetes, neurodegeneration, and cancer, thus prompting intense research to fully discern its regulatory modes and fuel the development of new therapeutic approaches [6–8].

In mammals, DNA methylation occurs at the fifth position of cytosine to produce 5-methylcytosine (5-mC). Maintenance of genomic methylation is ensured by the DNA methyltransferase DNMT1 which copies the DNA methylation pattern from the parental DNA strand onto the newly synthesized daughter strand during replication [9]. DNA methylation is also responsive and adaptive to environmental changes by modulating the expression of genes [10]. Two other methyltransferases, DNMT3A and 3B, have been described to methylate DNA de novo. Conversely, the ten–eleven translocation (TET) enzymes 1, 2, and 3 have the capacity to demethylate the genome [11–13].

The methyl group needed for DNA methylation is typically derived from dietary methionine, which is metabolized through the methionine cycle upon entering the cell. In this pathway, a molecule of ATP is transferred to methionine for S-adenosylmethionine (SAM) synthesis, the primary methyl donor for most biological methylation reactions [14]. After the methyl group is donated, the resulting S-adenosylhomocysteine (SAH) is either recycled or metabolized into cysteine, a nonessential amino acid that serves as a key building block for glutathione synthesis. The demethylation process catalyzed by the TET enzymes is also dependent on metabolite levels, and requires α-ketoglutarate (αKG), which can be generated from the tricarboxylic acid (TCA) cycle and glutaminolysis [12]. While DNA methylation and demethylation are clearly intrinsically linked to metabolism, the mechanisms that coordinate these programs remain poorly understood.

In the context of breast cancer (BC), several aspects of cellular metabolism are controlled by members of the estrogen related receptor (ERR) family [15]. The ERRs are orphan nuclear receptors that regulate a large variety of metabolic gene networks implicated in glycolysis, glutaminolysis, mitochondrial biogenesis, and cellular respiration [16, 17]. Recently, we have shown that ERRα is also a transcriptional regulator of the folate cycle, a metabolic pathway closely related to the methionine cycle [18]. ERRα also plays a central role in regulating the adaptive metabolic processes used by breast tumors to thrive in conditions of fluctuating nutrient availability [19]. Furthermore, high ERRα transcript levels are associated with the HER2 positive and triple-negative (TN) molecular subtypes known to be among the most aggressive forms of the disease [20].

In this study, we identify ERRα as a direct link between cellular metabolism and DNA methylation. We first show that inhibition of ERRα activity diminishes the expression of methionine cycle enzymes and markedly reduces DNMT1 transcription resulting in a global loss of cellular DNA methylation. In a feedforward regulatory loop, DNMT1 elevated ERRα protein, and levels of *DNMT1* mRNA correlated with high ERRα activity in BC patients. Importantly, pharmacological inhibition of ERRα further sensitized BC cells in vitro and in vivo to the anti-neoplastic effects of the DNMT inhibitor 5-aza-2′-deoxycytidine (decitabine, 5-AzadC). The clinical significance of our findings is further supported by genome-wide bisulfite sequencing, revealing that co-administration of ERRα and DNMT1 inhibitors leads to promoter demethylation and re-expression of *IRF4*-encoding Interferon Regulatory Factor-4 and found herein to exhibit tumor-suppressor activity in BC cells.

## Results

### ERRα regulates the expression of enzymes of the methionine cycle and DNA methylation

**Fig. 1:**
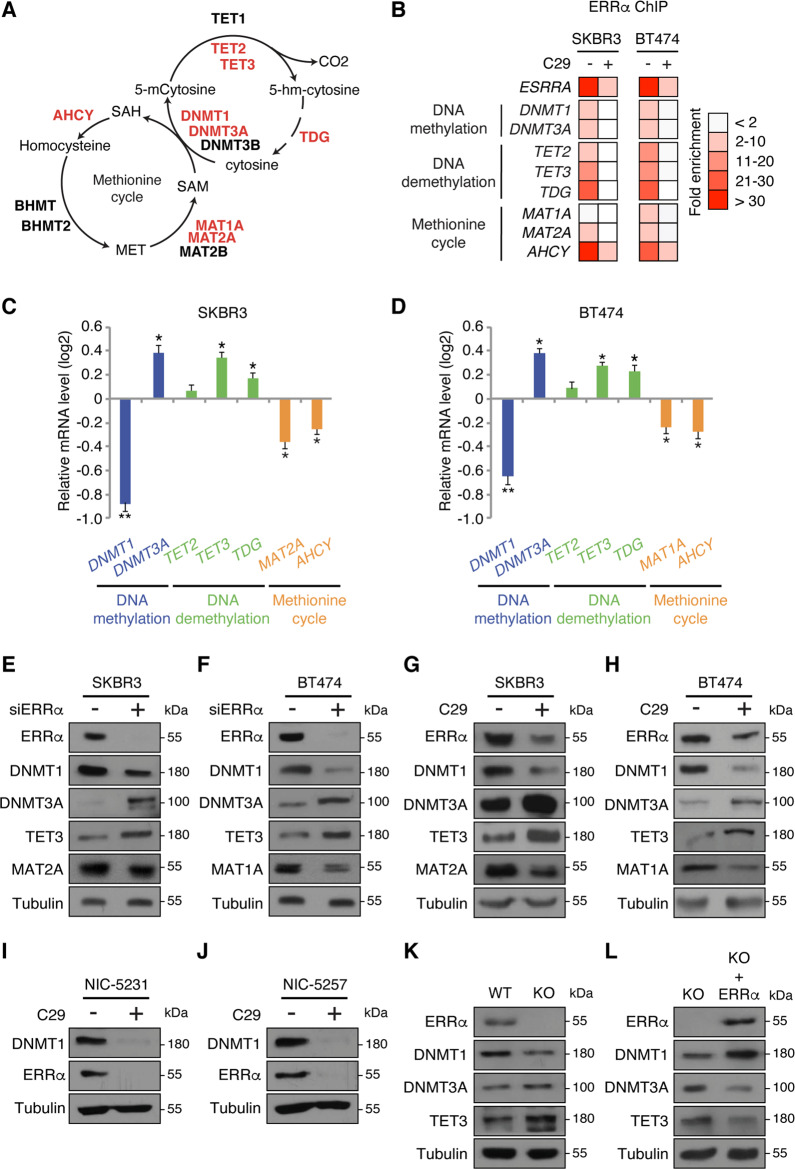
ERRα regulates genes involved in DNA methylation and the methionine cycle. **a** Schematic representation of the relationship between the methionine cycle and DNA methylation. Enzymes in red were identified as direct ERRα target genes by ChIP-seq. **b** ChIP-qPCR analysis of ERRα binding on the target genes identified in (**a**) following treatment with C29 for 24 h (*n* = 3). **c**, **d** qRT-PCR analysis of SKBR3 (**c**) and BT474 (**d)** cells after treatment with C29 for 24 h. Control cells treated with vehicle were set at 0. Results reflect three independent experiments performed in triplicate. **e**, **f** Immunoblot analysis of SKBR3 (**e**) and BT474 (**f**) cells post-transfection for 48 h with siRNAs against ERRα. Tubulin is shown as a loading control. **g**, **h** Immunoblot analysis of SKBR3 (**g**) and BT474 (**h**) cells following treatment with C29 for 24 h. Tubulin is shown as a loading control. **i**, **j** Immunoblots of ERRα and DNMT1 in the mouse BC cell lines NIC-5231 (**i**) and NIC-5257 (**j**) after treatment with C29 for 24 h. Tubulin is shown as a loading control. **k** Immunoblots of ERRα KO MEFs compared to WT. Tubulin is shown as a loading control. **l** Immunoblots of ERRα KO MEFs with ectopic expression of ERRα compared to the parental KO MEFs. Tubulin is shown as a loading control. Data shown in **c** and **d** represent means ± SEM. *p < 0.05, **p < 0.01; Student’s *t* test.

DNA methylation is dependent on cellular metabolic activity, specifically the methionine cycle (Fig. 1a), and considering that the ERRs are key transcriptional regulators of cell metabolism, we investigated whether ERRα is directly implicated in this process. To this end, we interrogated ERRα ChIP-seq datasets obtained in the BC cell lines BT474 and SKBR3 [21]. These cells are characterized by high expression of the receptor tyrosine kinase HER2, known to drive ERRα function [22, 23]. Consequently, BT474 and SKBR3 cells possess high ERRα activity and represent ideal models for our investigation. ERRα-binding sites were found near the transcriptional start sites of the DNA methytransferase genes *DNMT1* and *DNMT3A*, the DNA demethylation enzyme genes *TET2*, *TET3*, and *TDG*, and the *AHCY* gene of the methionine cycle in both cell lines (Supplementary Fig. 1a). ERRα was found specifically bound to regulatory regions near *MAT1A* in BT474 cells and *MAT2A* in SKBR3 cells suggesting cell-specific differences in isoform expression (Supplementary Fig. 1a). ChIP-qPCR validated the recruitment of ERRα to these sites which was lost when cells were treated with the specific ERRα inhibitor C29 [24] (Fig. 1b). ERRα inhibition with C29 led to a significant induction of the DNA-demethylating genes *TET3* and *TDG* along with an observed inverse regulation of DNA methylating genes with downregulation of *DNMT1* and upregulation of *DNMT3A* (Fig. 1c, d). Further, targeting ERRα diminished the expression of *AHCY*, as well as that of *MAT1A* and *MAT2A*, respectively, in BT474 and SKBR3 cells (Fig. 1c, d), the latter in line with ERRα ChIP-seq binding profiles. Immunoblot analysis confirmed similar effects on the protein levels of these genes following ERRα knockdown or inhibition by C29 in both HER2+ cell lines (Fig. 1e–h). Although, HER2 can positively regulate ERRα activity, ERRα is also expressed in the ER+ and TN BC subtypes. To verify whether ERRα regulation of DNA methylating enzyme expression is subtype-specific, we looked at protein levels of DNMT1 after knockdown of ERRα by RNA interference in MCF7 cells, an ER+ BC cell line, as well as in the three TNBC cell lines MDA-MB-231, MDA-MB-436, and MDA-MB-468. In each case, impairment of ERRα function reduced DNMT1 protein levels (Supplementary Fig. 1b–e).

Importantly, this specific involvement of ERRα in the regulation of DNA methylation is not restricted to human cancer cells. Drug-induced inhibition of ERRα by C29 in the mouse cell lines NIC-5231 and NIC-5257, derived from ErbB2-driven mammary tumors [25], also led to a stark reduction in DNMT1 protein (Fig. 1i, j). This mechanism is also conserved in normal cells whereby ERRα knockout mouse embryonic fibroblasts (ERRα KO MEFs) exhibited similar alterations in DNMT isoforms and TET expression as compared to BC cells, a phenotype reversed by ectopic expression of ERRα (Fig. 1k, l).

### ERRα controls DNA methylation

**Fig. 2:**
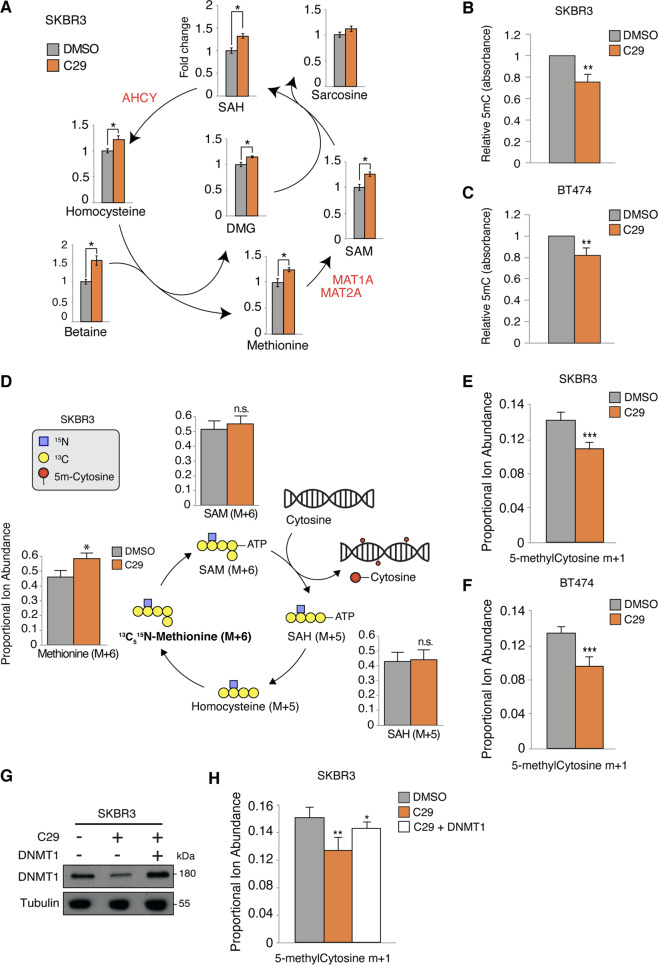
ERRα is a driver of DNA methylation. **a** Relative level of metabolites of the methionine cycle upon treatment with C29 for 24 h in SKBR3 cells. Results reflect three independent experiments each performed with five replicates. **b**, **c** Relative 5-methylcytosine levels in SKBR3 (**b**) and BT474 (**c**) cells after treatment with C29 for 24 h. Results reflect three independent experiments performed in triplicate. **d** Stable isotope tracing diagram for ^13^C_5_^15^N-methionine through the methionine cycle and DNA methylation. Levels of measured labeled methionine cycle intermediates in SKBR3 cells reflect three independent experiments each performed with five replicates. **e**, **f** Relative quantification of DNA labeled 5-methylcystosine (m + 1) after treatment with C29 in the presence of ^13^C_5_^15^N-methionine for 24 h in SKBR3 (**e**) and BT474 (**f**) cells. Results reflect three independent experiments each performed with five replicates. **g** DNMT1 protein expression in SKBR3 cells after treatment with C29 for 24 h ± ectopic expression of DNMT1. Tubulin is shown as a loading control. **h** Relative quantification of DNA labeled 5-methylcystosine (m + 1) in SKBR3 cells after treatment with C29 ± ectopic expression of DNMT1 in the presence of ^13^C_5_^15^N-methionine for 24 h. Results reflect three independent experiments each performed with five replicates. Data shown in **a**–**f** and **h** represent means ± SEM. *p < 0.05, **p < 0.01, ***p < 0.001; Student’s *t* test.

To investigate the influence of ERRα on the methionine cycle and DNA methylation programs, we measured the steady-state levels of methionine cycle intermediates in SKBR3 cells after pharmacological inhibition of ERRα with C29 for 24 h. Impeding ERRα resulted in a significant accumulation of all intermediates of this metabolic process (Fig. 2a). Moreover, C29-mediated ERRα inhibition led to a marked decrease in global DNA methylation in both SKBR3 and BT474 cell lines, exemplified by a significant reduction in total 5-methylcytosine levels (Fig. 2b, c). Importantly, ERRα KO MEFs also displayed a decreased level of DNA methylation compared to WT MEFs (Supplementary Fig. 2).

The accumulation of methionine cycle intermediates might be the consequence of a reduced metabolic rate due to the decreased expression of the methionine cycle enzymes we observed (Fig. 1c–h) or a bottleneck downstream in the transfer of a methyl group from SAM to DNA. To measure the metabolic rate of the methionine cycle, we designed an isotope tracer experiment whereby SKBR3 cells were first treated with C29 for 24 h, followed by incubation with labeled methionine (^13^C_5_^15^N-methionine) for 2 h. Given that methionine is an essential amino acid, we could follow the incorporation of labeled atoms from dietary methionine into SAM, SAH, and homocysteine by liquid chromatography coupled to mass spectrometry (LC/MS). Interestingly, while we could not detect homocysteine in this setting, C29-treated cells displayed an increase of labeled methionine (Fig. 2d). Of note, C29 had no significant impact on SAM and SAH levels, suggesting a similar rate of the methionine cycle compared to control (Fig. 2d).

We then quantified the levels of labeled 5-methylcytosine arising from labeled methionine. BC cells were first treated with C29 for 24 h and then incubated with labeled methionine for another 24 h to allow for labeled methyl incorporation prior to genomic DNA isolation. As expected, diminished levels of labeled 5-methylcytosine were observed when ERRα activity was impaired (Fig. 2e, f). Notably, this effect was largely rescued by exogenous expression of DNMT1 (Fig. 2g, h), highlighting that DNMT1 is the rate-limiting enzyme and is critical for driving DNA methylation in this context.

### ERRα activity correlates with *DNMT1* expression in BC patients

**Fig. 3:**
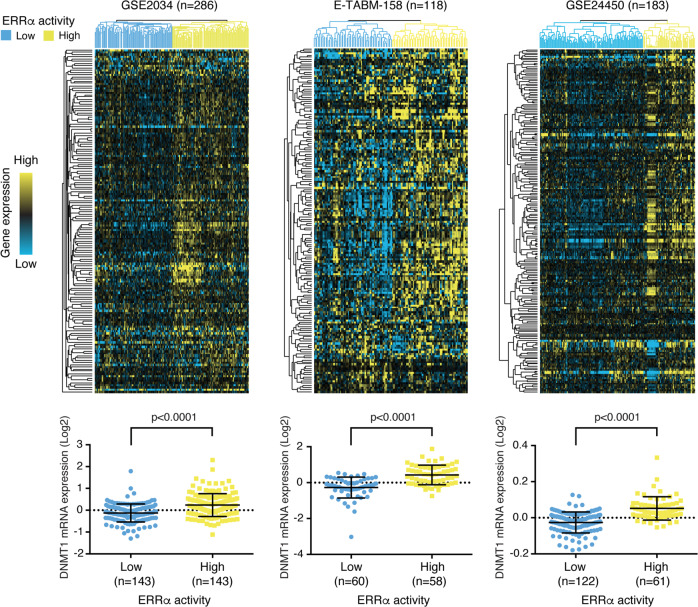
ERRα activity positively correlates with DNMT1 expression. Top, unsupervised hierarchical clustering analysis with an ERRα-targeted gene signature in three independent BC clinical cohorts. Subtype colors discriminate between patients with low ERRα activity (blue) and patients with high ERRα activity (yellow). Bottom, relative *DNMT1* mRNA levels between patients with low and high ERRα activity for each BC cohort. *DNMT1* expression values were extracted from microarray data after normalization and were log2 transformed. Data show means ± SEM; Student’s *t* test.

We next re-analyzed publicly available gene expression datasets from cohorts of BC patient tumors of mixed molecular subtypes to determine whether ERRα activity correlates with the expression of DNA methylation regulators. For this, we utilized a previously established 121-gene ERRα signature shown to cluster BC patients into groups of low or high ERRα activity independent of their BC molecular subtype [23]. To this gene list, we added key ERRα-targeted genes identified in this study namely *DNMT1, DNMT3A, TET2, TET3, AHCY, MAT1A*, and *MAT2A*, as they were not included in the original dataset (Supplementary Table 1). Unsupervised hierarchical clustering successfully partitioned the tumor profiles into 2 groups distinguished by having either low or high ERRα activity across three independent cohorts obtained from Gene Omnibus and ArrayExpress (GSE2034, GSE24450 and E-TABM-158), thus confirming the validity of the ERRα signature (Fig. 3). Next, we tested for a significant association between the expression of our genes of interest and ERRα activity. Of the genes examined, only *DNMT1* transcript levels showed a consistent and significant correlation with BC tumors bearing high ERRα activity across the three independent patient cohorts (Fig. 3 and Supplementary Fig. 3). This raises the possibility that simultaneous inhibition of DNMT1 and ERRα activity may offer a therapeutic advantage for the treatment of BC patients.

### Dual inhibition of ERRα and DNMT suppresses BC cell growth in vitro

**Fig. 4:**
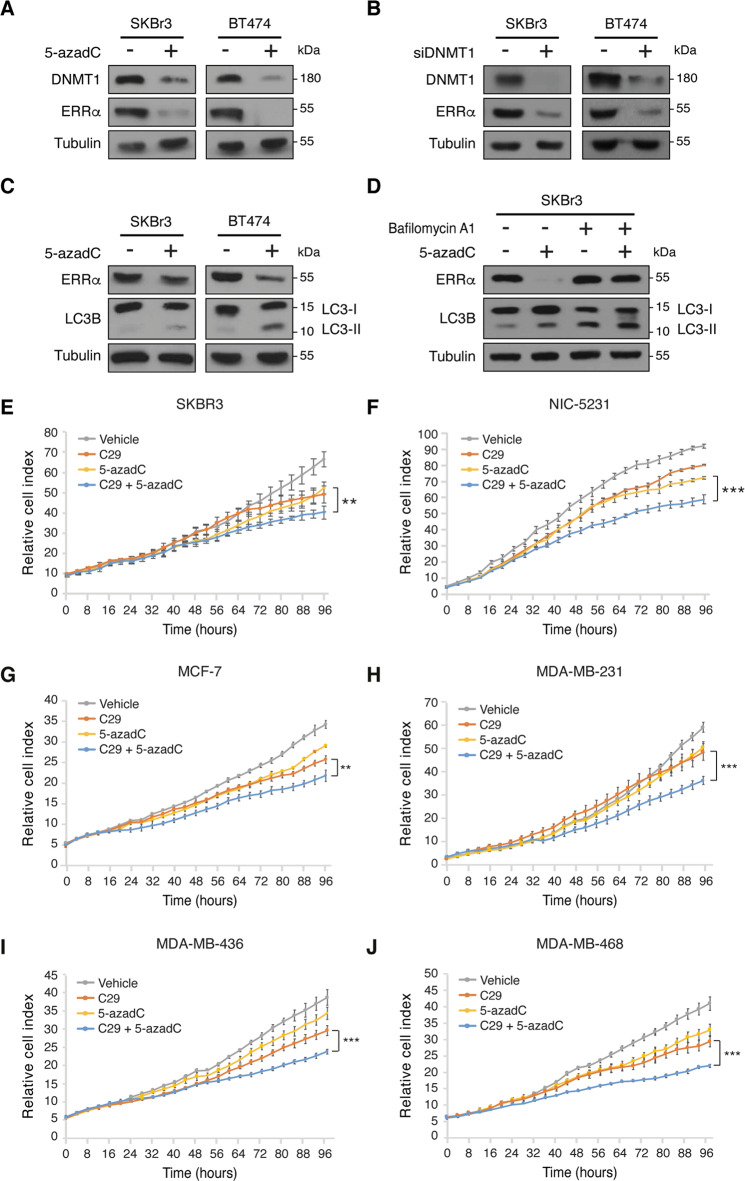
Dual inhibition of ERRα and DNMT augments BC cell growth hindrance in vitro. **a** Immunoblots of ERRα and DNMT1 after DNMT1 inhibition by treatment with the DNMT inhibitor 5-azadC (5 μM) for 24 h in both SKBR3 and BT474 cells. Tubulin is shown as a loading control. **b** Immunoblots of ERRα and DNMT1 post-transfection with siRNAs against DNMT1 for 48 h in both SKBR3 and BT474 cells. Tubulin is shown as a loading control. **c** Immunoblot of ERRα and the marker of autophagy LC3B in SKBR3 and BT474 cells after treatment with 5 μM 5-azadC for 24 h. **d** Protein levels of ERRα and LC3B in SKBR3 cells after treatment with 5 μM 5-azadC for 24 h in combination with 20 nM of the autophagy inhibitor Bafilomycin A1. Bafilomycin A1 was added 6 h prior harvesting cells. **e**–**j** Normalized cell index curves representing proliferation of human HER2+ SKBR3 (**e**), mouse HER2+ NIC-5231 (**f**), human ER+ MCF7 (**g**), TNBC MDA-MB-231 (**h**), TNBC MDA-MB-436 (**i**), and TNBC MDA-MB-468 (**j**) cells in the presence of C29 (5 μM) and/or 5-azadC (3 μM). Three independent experiments each with five replicates were performed and one representative experiment is shown. Data shown in **e**–**j** represent means ± SEM. **p < 0.01, ***p < 0.001; Student’s *t* test.

While human cancer cells generally harbor global DNA hypomethylation profiles, they also specifically display hypermethylation of promoters of tumor suppressor genes leading to their silencing [26]. Accordingly, inhibition of DNMTs correlates with reduced tumorigenicity often related to re-expression of tumor suppressors [27]. Recently, the FDA-approved DNMT inhibitor 5-azadC (decitabine) was proven effective in treating TNBC [28]. Given our identification of an association between ERRα activity and *DNMT1* expression in BC patients, we tested whether ERRα inhibition could further sensitize BC cells to 5-azadC. Unexpectedly, 5-azadC alone robustly reduced ERRα protein levels, an effect also observed following the specific knockdown of DNMT1 by RNA interference in both SKBR3 and BT474 cells (Fig. 4a, b). Further analysis determined that the DNA-demethylating agent 5-azadC induces BC cell autophagy, as indicated by increased levels of the autophagy marker LC3B-II (Fig. 4c), and that blockade of autophagy with bafilomycin A is sufficient to rescue 5-azadC-induced ERRα degradation (Fig. 4d). 5-azadC has also been shown to promote autophagy in ovarian cancer cells [29].

Next, we examined the effect of co-treatment with the ERRα inhibitor C29 and 5-azadC on BC cellular proliferation. While 5-azadC alone significantly diminished the growth of both the HER2 human SKBR3 and mouse NIC-5231 cell lines, C29 amplified the neoplastic effects of the DNMT inhibitor (Fig. 4e, f). Similar beneficial anti-tumor effects of combined ERRα and DNMT inhibitors were observed in the ER+ BC cell line MCF7 and TNBC cell lines MDA-MB-231, MDA-MB-436, and MDA-MB-468 (Fig. 4g–j and Supplementary Fig. 4a–d).

Since ERRα activity is regulated by growth factors and HER2 [30], we wondered whether classical anti-HER2 therapy would also sensitize SKBR3 cells to the DNMT inhibitor 5-azadC. Similar to ERRα loss-of-function in BC cells (Fig. 1e–j), SKBR3 cells treated with lapatinib, a dual epidermal growth factor receptor (EGFR)/human EGFR-2 (HER2) tyrosine kinase inhibitor approved for patients with HER2-amplified breast tumors, decreased both ERRα and DNMT1 protein levels (Supplementary Fig. 4e). However, lapatinib was also found to reduce DNMT3a levels (Supplementary Fig. 4e), while ERRα inhibition seemingly stabilized it (Fig. 1e–h, k). Co-treatment of SKBR3 cells with lapatinib and 5-azadC did not further increase the anti-tumoral effects of each drug alone (Supplementary Fig. 4f), suggesting that the effects of anti-HER2 therapy beyond ERRα inhibition impede the benefit of co-targeting ERRα and DNMT simultaneously.

### C29 enhances the efficacy of 5-azadC on impeding BC tumor development in vivo

**Fig. 5:**
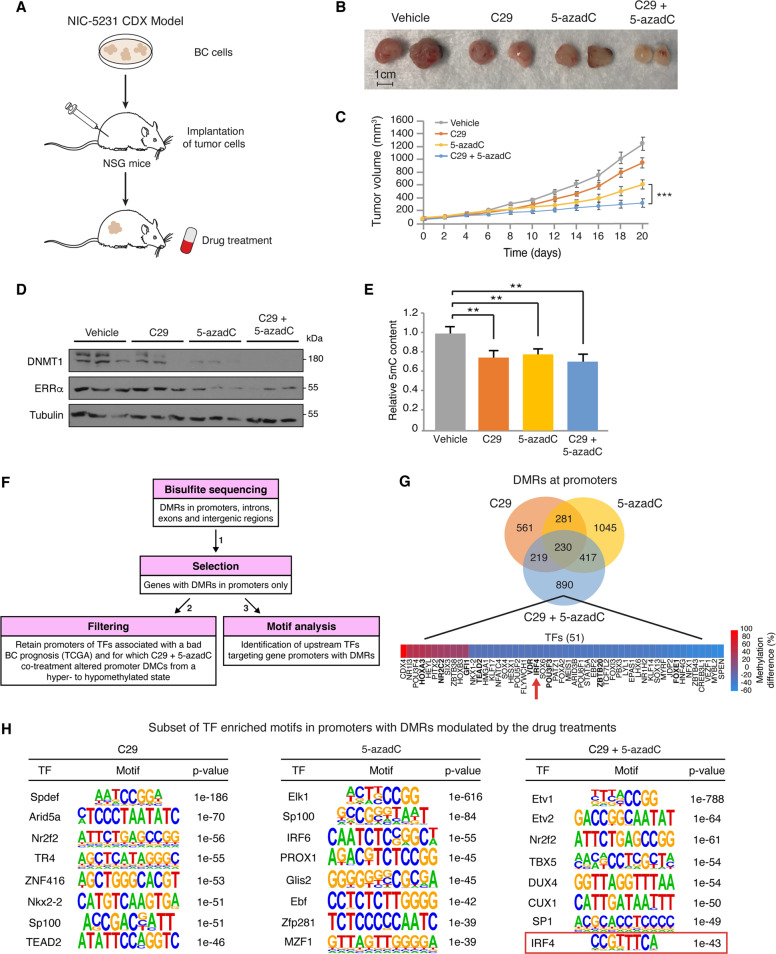
Decitabine and C29 act in concert to suppress BC tumor growth in vivo. **a** Schematic illustrating the establishment of a mouse BC CDX model from NIC-5231 cells for pharmacological drug testing in a pre-clinical setting. **b** Representative images of tumors extracted at endpoint (20 days post-treatment) from mouse mammary fat pads illustrating the effect of the different drug regimens on tumor size. **c** Tumor growth curves following administration of C29 (10 mg/kg), 5-azadC (1 mg/kg) or a combination of both drugs (*n* = 5 for each group). **d** Immunoblots of DNMT1 and ERRα in tumors after a 20-day drug regimen (*n* = 3 samples per group are shown). Tubulin is shown as a loading control. **e** Relative quantification of 5-methylcytosine levels in tumors after 20 days of treatment (*n* = 5 per group). **f** Computational pipeline developed for the identification of genes of interest from the RRBS analysis on tumors of mice treated with the different drug regimens. **g** Venn diagram representing the overlap of the number of DMRs in gene promoters after treatment with C29, 5-azadC or a combination of both drugs. A heatmap of the 51 transcription factors found with differential promoter methylation after C29 and 5-azadC co-treatment is shown. Red represents promoter hypermethylation, while blue designates promoter hypomethylation. The red arrow points at the transcription factor IRF4. **h** Subset of TF motif enrichment analysis performed on the total list of DMRs identified following treatment with C29, 5-azadC or a combination of both drugs. The IRF4 motif (boxed in red) was specifically enriched in promoters with DMRs of tumors co-treated with C29 + 5-azadC. Data shown in (**c**) and (**e**) represent means ± SEM. **p < 0.01, ***p < 0.001; Student’s *t* test.

To determine the efficacy of the therapeutic agent 5-azadC in combination with C29 at suppressing BC tumor growth in vivo, compared to 5-azadC therapy alone, we used a pre-clinical mouse cell line-derived xenograft (CDX) model. NIC-5231 cells were injected into the mammary fat pad of NSG mice, and primary tumors were treated with either C29 (10 mg/kg) and/or 5-azadC (1 mg/kg), or vehicle control (Fig. 5a). As anticipated, treatment with the DNA-demethylating drug 5-azadC alone significantly attenuated tumor growth (Fig. 5b, c). Importantly, as observed in vitro, C29 potentiated the tumor suppressive effect of 5-azadC in vivo, thus validating the utility of combining ERRα and DNA methyltransferase inhibitors in the pharmacological intervention of BC (Fig. 5b, c). Immunoblot analysis of end-point tumors confirmed the positive molecular link between ERRα and DNMT1 expression whereby ERRα loss of function by C29 decreased DNMT1 protein and treatment with 5-azadC reduced ERRα levels (Fig. 5d). Surprisingly, while C29 and 5-azadC independently decreased intra-tumoral levels of 5-methylcytosine, there was no further significant decline in global DNA methylation upon co-treatment compared to the individual drug regimens (Fig. 5e). We therefore hypothesized that C29 and 5-azadC may have differential effects on promoter-specific DNA methylation that may be otherwise masked by the evaluation of global DNA methylation states. To address this, we performed reduced representation bisulfite sequencing (RRBS) on tumor DNA from these mice. RRBS is a high-throughput technique that offers a large-scale high-resolution mapping of DNA methylation across the genome that enriches for regions with high CpG content such as promoters and repeated sequences [31]. Overall, lower methylated CpGs were found in all three drug treatment groups compared to control, and the repartitions of the differentially methylated CpGs between introns, exons, promoters and intergenic regions were almost identical across treatment groups (Supplementary Fig. 5a–f). Pathway enrichment analysis of the genes with differentially methylated regions (DMRs) showed no major differences between treatment groups (Supplementary Fig. 5g–i). We next devised a pipeline as outlined in Fig. 5f to focus our attention on more precise features of the tumor RRBS datasets using several filtering criteria. First, since cancer cells possess specific promoter hypermethylation of tumor suppressor genes, we restricted our analysis to DMRs located within promoter regions (Supplementary Table 2). Given that promoters are under the control of TFs, we looked specifically for TFs targeting these regions and for which C29 and 5-azadC co-treatment induced promoter hypomethylation. Promoter methylation status was analyzed in silico using the SMARTapp [32], which allowed us to identify TFs possessing hypermethylated CpG sites that correlate with bad prognosis in BC patients. Those presenting hypomethylated CpG sites following the combinatorial drug regimen were retained, given that they offer a therapeutic benefit by allowing for re-expression of tumor suppressor genes. Third, to identify candidate TFs regulating these genes, Homer (Hypergeometric Optimization of Motif EnRichment) software was used for analysis of TF motif enrichment on gene promoters harboring DMRs [33]. In our analysis, 890 genes with promoter DMRs were found uniquely modified in tumors co-treated with C29 and 5-azadC—this included 51 TFs based on a recent article referencing all known human TFs (Fig. 5g and Supplementary Table 3) [34]. Among these 51 TFs, 9 were characterized in the SMARTapp as having promoter CpG hypermethylation and a significant association with bad prognosis in BC. Dual inhibition of ERRα and DNMT specifically induced promoter hypomethylation of six of these nine prognostic TFs: IRF4, FOXE1, VDR, TEAD2, POU3F3, and ZBTB20 (Fig. 5g and Supplementary Table 3). Both VDR and ZBTB20 were previously shown to operate as tumor suppressors in cancer [35, 36]. IRF4 has also been found to have both oncogenic and tumor suppressive activities in hematological cancer [37, 38]. Further examination with TF motif enrichment analysis revealed strong differences in potential TFs implicated in the response to each treatment (Fig. 5h). Intriguingly, the IRF4 motif was specifically enriched after co-treatment with C29 and 5-azadC (Fig. 5h), a condition found to demethylate the *IRF4* promoter (Fig. 5g). Thus, the computational analysis highlights IRF4 as a potential active participant in mediating the anti-tumor effects observed. Alongside this implication, we noted that the strongest motif enriched in the combined treatment group was for ETV1, a member of the ETS family of TFs and well-known as an oncogene in several cancer types including BC [39]. Considering that a role for IRF4 in BC has never been explored, we next sought to investigate its potential tumor suppressor activity in this context.

### Combined C29 and 5-azadC treatment reverses *IRF4* promoter hypermethylation in BC

**Fig. 6:**
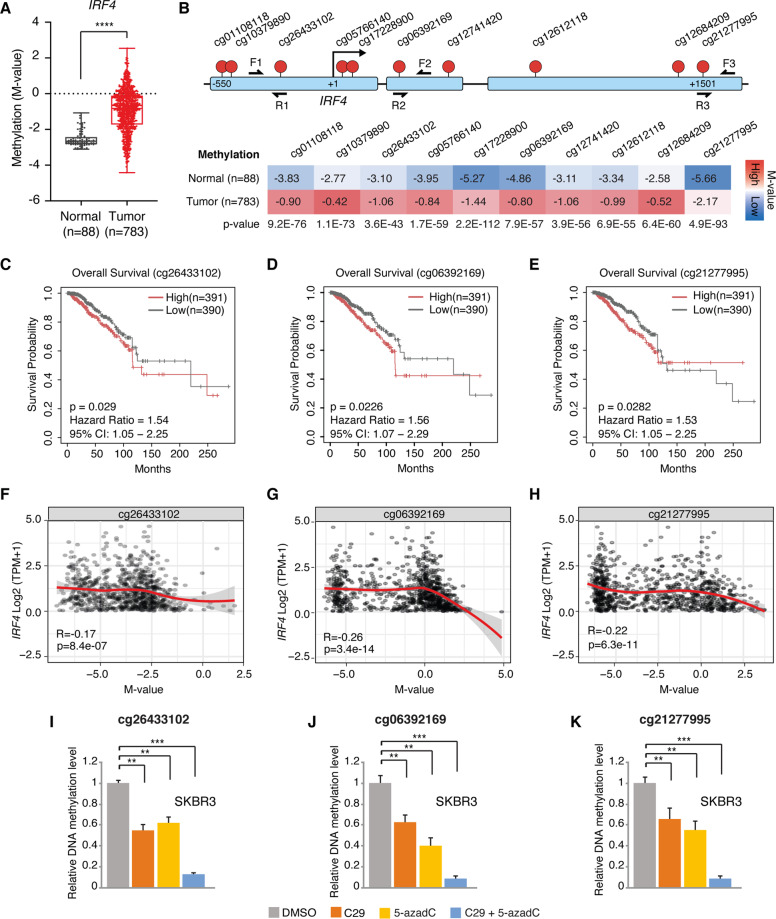
The *IRF4* promoter is hypermethylated in BC patients and correlates with poor overall survival. **a** Box plot showing the methylation status of *IRF4* in BC patients using the mean aggregation of all 16 CpGs referenced within the SMARTapp obtained from the TCGA project. The outer limits of the box represent the 25th (lower quartile) and 75th percentile (upper quartile) with the median value shown inside. Whiskers extend to the lowest and highest values. ****p < 0.0001; Student’s *t* test. **b** Schematic representation of the *IRF4* promoter containing ten CpG sites identified by SMARTapp found within three CpG islands (blue bars). The corresponding relative mean methylation levels for each of the 10 promoter-specific CpG sites in normal tissue and BC tumor samples (*M*-value) are shown. The associated *p* values were calculated using the Student’s *t* test. The pairs of primers used for MS-qPCR analysis in **i**–**k** are represented as F1-R1 for cg26433102, F2-R2 for cg06392169, and F3-R3 for cg21277995. **c**–**e** Kaplan–Meier survival curves derived from the SMARTapp illustrating the correlation between the methylation status (*M*-value) of the *IRF4*-associated CpG sites cg26433102 (**c**), cg06392169 (**d**), and cg21277995 (**e**) with BC patient overall survival. **f**–**h** Spearman correlation curves obtained from the SMARTapp showing the association between the methylation status (*M*-value) of the *IRF4*-associated CpG sites cg26433102 (**f**), cg06392169 (**g**), and cg21277995 (**h**) and *IRF4* gene-level expression in BC patients (*n* = 853). **i**–**k** Relative methylation levels of the *IRF4*-associated CpG sites cg26433102 (**i**), cg06392169 (**j**), and cg21277995 (**k**) after treatment with C29 (5 μM), 5-azadC (3 μM) or a combination of both drugs for 7 days in SKBR3 cells. Data presented in (**i**–**k**) show means ± SEM. **p < 0.01, ***p < 0.001; Student’s *t* test.

We first aimed to confirm in silico the methylation status of *IRF4* in cancer. The SMARTapp revealed the existence of 16 CpGs with available methylation data for this gene. Aggregating the mean methylation levels of all CpGs, *IRF4* is significantly hypermethylated in BC as well as in almost every other cancer type within the TCGA collection (Fig. 6a and Supplementary Fig. 6a). Specifically, 10 out of 16 CpGs are localized in the *IRF4* promoter (Fig. 6b). According to SMARTapp, these ten sites are all hypermethylated in BC with nine displaying a significant positive correlation with poor overall survival (Fig. 6b–e and Supplementary Fig. 6b). Moreover, their methylation status negatively correlated with gene-level expression, confirming that hypermethylation of the *IRF4* promoter would lead to *IRF4* silencing (Fig. 6f–h and Supplementary Fig. 6c). To validate this, we selected one CpG site within each of the three CpG islands found within the *IRF4* promoter (Fig. 6b), and performed methylation-specific quantitative PCR (MS-qPCR), a technique that allows direct evaluation of the methylation status of a specific CpG site [40]. Compared to control SKBR3 cells treated with vehicle, the level of methylation at all three CpG sites tested was significantly reduced by the addition of either C29 or 5-azadC. Remarkably, these effects were additive, as co-treatment of the drugs effectively abrogated *IRF4* promoter CpG methylation (Fig. 6i–k).

### The suppressive effects of concomitant ERRα and DMNT inhibition on BC growth is dependent on *IRF4* derepression

**Fig. 7:**
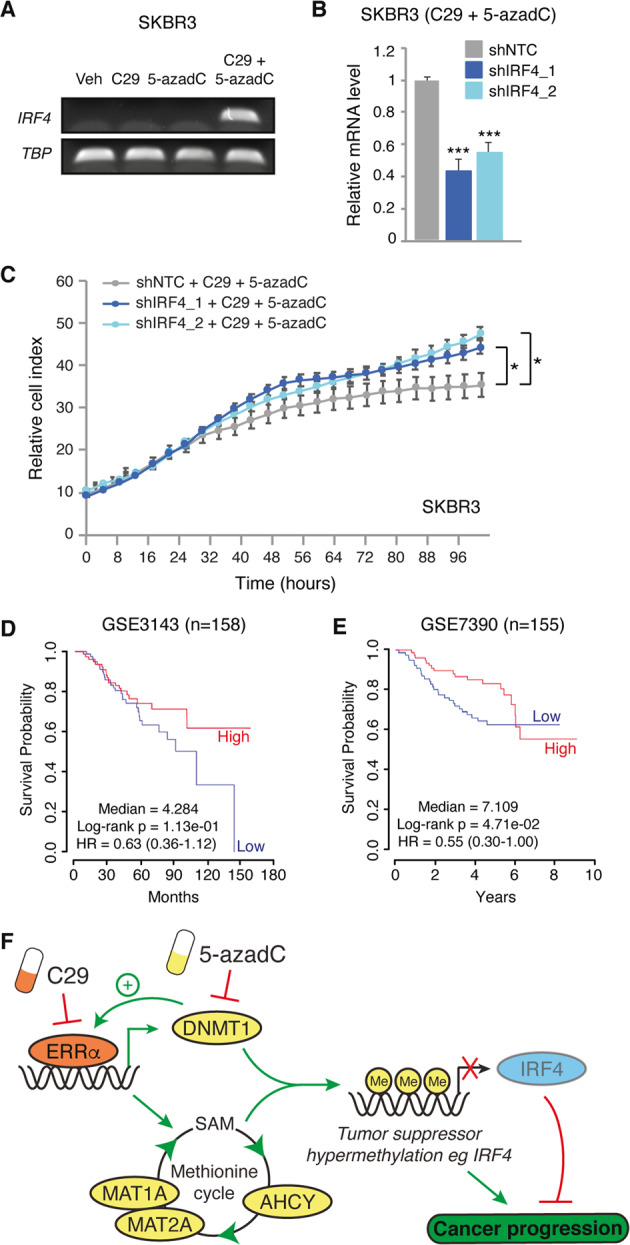
IRF4 is a tumor suppressor in BC re-expressed upon co-treatment with C29 and 5-azadC. **a** Agarose gel electrophoresis showing PCR product amplification of IRF4 cDNA following treatment with C29 (5 μM), 5-azadC (3 μM) or a combination of both drugs for 7 days in SKBR3 cells. **b** Relative mRNA levels of *IRF4* in SKBR3 cells infected with shRNAs against IFR4 after treatment with C29 (5 μM), 5-azadC (3 μM) or a combination of both drugs for 7 days. **c** Normalized cell index curves representing proliferation of SKBR3 cells infected with either a control shRNA (shNTC) or two different shRNAs against IRF4 in the presence of C29 (5 μM) and 5-azadC (3 μM). Data represent one experiment performed with five replicates. **d**, **e** Kaplan–Meier survival curves representing the positive correlation between *IRF4* mRNA expression and overall survival of BC patients in two independent cohorts consisting of 158 patients (GSE3143) (**d**) and 155 patients (GSE7390) (**e**). **f** Model illustrating how pharmacological inhibition of the inter-connected factors ERRα and DNMT1 can halt BC progression by simultaneously repressing methionine cycle metabolism and DNA methylation. Consequent epigenetic modulation impinges on the newly attributed tumor suppressor gene IRF4 in BC with C29 and 5-azadC co-treatment promoting IRF4 derepression. Data shown in **b** and **c** represent means ± SEM. *p < 0.05, ***p < 0.001; Student’s *t* test.

To verify *IRF4* expression, we performed RT-PCR on SKBR3 cells treated with C29 and/or 5-azadC, or vehicle control, using primers designed to amplify a 200 bp cDNA region of *IRF4*. In accordance with the state of *IRF4* promoter methylation (Fig. 6i–k), we could not detect *IRF4* in SKBR3 cells under basal conditions or in response to C29 or 5-azadC treatment alone (Fig. 7a). However, co-administration of both drugs resulted in amplification of the *IRF4* cDNA (Fig. 7a), thus confirming that concurrent inhibition of ERRα and DMNT can successfully derepress the *IRF4* gene that was silenced by promoter hypermethylation. We next sought to provide evidence for a tumor suppressive action of *IRF4* re-activation underlying the therapeutic benefit to a combined ERRα and DNMT drug therapy in BC. Accordingly, SKBR3 cells were first infected with either a non-specific shRNA (shNTC) or with one of two different shRNAs targeting *IRF4* (Fig. 7b). Subsequently, the cells were exposed to either a combined C29 and 5-azadC drug regimen or vehicle control and the impact on cellular growth was evaluated (Fig. 7c and Supplementary Fig. 7). As expected, knockdown of *IRF4* had no impact on cell growth in the vehicle condition, a context in which *IRF4* is already silenced (Supplementary Fig. 7). In stark contrast, while C29 and 5-azadC inhibited cell proliferation, this effect was demonstrated to be IRF4-dependent as shRNA-mediated suppression of *IRF4* could significantly restore cell growth capabilities, underscoring an anti-proliferative function of IRF4 in BC cells (Fig. 7c). Finally, we interrogated the PRECOG website (https://precog.stanford.edu/) [41] to investigate the correlation between *IRF4* expression and patient overall survival in BC. Our analysis confirmed a positive correlation between *IRF4* expression and favorable patient outcome, thus validating our findings that IRF4 plays a tumor suppressor role in BC (Fig. 7d, e).

## Discussion

In this study, we identified a new ERRα-dependent regulatory pathway conserved across species linking cell metabolism and DNA methylation. We show that ERRα, a major regulator of cellular metabolism, coordinates SAM synthesis through the methionine cycle while driving DNMT1 expression to promote DNA methylation. Genetic or pharmacological inhibition of ERRα repressed DNMT1 expression, the activity of the methionine cycle and, ultimately, global DNA methylation. Reciprocally, we uncovered that inhibiting DNMT1 diminishes ERRα levels, suggesting that DNMT1 directly influences cell metabolism. ERRα activity and DNMT1 expression were found to positively correlate in BC patients independent of BC subtype, reinforcing the molecular link between these two genomic regulators. Of clinical relevance, targeting ERRα with the specific inhibitor C29 significantly increased the sensitivity of BC cells to the DNMT inhibitor decitabine both in vitro and in vivo. A large-scale analysis of DNA methylation further revealed that co-treatment with both drugs alters promoter methylation of a specific set of genes, leading to the identification and functional characterization of *IRF4*, found to possess tumor suppressor activity in BC and derepressed in this context.

While DNA methylation is tightly bound to the metabolic state of the cell, active DNA demethylation also occurs and depends on the availability of specific metabolites. Indeed, we observed that ERRα inhibition induced the expression of the demethylase TET3, suggesting that ERRα could also be involved in active DNA demethylation (Fig. 1). However, we speculate that loss of DNA methylation linked to ERRα inhibition is more likely due to a lack of DNMT1 activity than to an increase in demethylase activity. This hypothesis is supported by our recent report demonstrating that ERRα inhibition depletes the available pool of αKG [21], a required cofactor of dioxygenase enzymes such as the TET DNA demethylases. αKG is mostly synthesized through glutaminolysis in cancer, a pathway regulated by ERRα in BC [42], and is an important intermediate of the TCA cycle involved in many cellular functions such as anti-oxidation, protein, and lipid synthesis, as well as cellular respiration [43].

The methionine cycle is central to several essential metabolic pathways. Notably, methionine combines with ATP to produce SAM, the principal methyl donor for methylation of proteins, DNA, RNA, and lipids [44]. By donating one carbon for methylation, SAM generates SAH, the precursor of homocysteine which can ultimately produce glutathione, the main cellular antioxidant [45]. However, regeneration of methionine from homocysteine has been proven to be low in cancer cells, rendering them highly dependent on exogenous methionine [14, 46]. Hence, dietary methionine restriction is under intense investigation as a potential anti-cancer therapy and has shown promising results in improving metabolism, increasing lifespan and preventing cancer cell growth in numerous contexts [47–52]. Given the clear importance of methionine in cancer, the mechanisms through which methionine cycle enzymes are regulated must be well understood. Here, we show that ERRα positively regulates the expression of several methionine cycle genes, influencing the levels of intermediate metabolites. These findings are significant in the light of our recent work showing that ERRα represses the folate cycle and that ERRα inhibition leads to an increase in purine biosynthesis [18]. Considering the intimate relationship between these two pathways as the recycling of methionine links the methionine and the folate cycles, nucleotide synthesis and NADH/NADPH production, it raises the question as to why ERRα would regulate these programs differently [53–55]. One possible reason stems from a recent study demonstrating that the methionine and folate cycles compete for metabolites involved in DNA methylation, nucleotide synthesis, and anti-oxidation [56]. This suggests that ERRα might act as a switch or sensor to balance these processes [57], which could be particularly important in cancer cells where high nucleotide synthesis, elevated oxidative states, and DNA hypomethylation are often observed.

Cancer cell DNA is characterized by promoter hypermethylation of tumor suppressor genes that induces their silencing. Thus, demethylating agents such as 5-azadC have been proposed as anti-cancer therapies with the intention of re-establishing tumor suppressor expression. 5-azadC is currently used clinically for the treatment of myelodysplastic syndrome and other leukemias, where the drug has received FDA approval [58]. However, clinical development of this drug is still prohibited in solid tumors due to substantial toxicity [59]. Nevertheless, anti-tumor activity of 5-azadC has been reported in BC patients with a response rate of up to 50% and, more recently, levels of DNMT proteins have been proposed as biomarkers for decitabine response in TN BC [28, 60]. These observations suggest that a better understanding of the mechanisms of regulation of DNA methylation in BC might help to improve the use of demethylating agents in anti-cancer regimens. Here, our work shows DNMT1 supporting ERRα as a driver of DNA methylation to fuel BC development, thus highlighting a therapeutic advantage of targeting both factors (Fig. 7f). We have shown that the combinatorial drug therapy induces a new anti-tumor mechanism involving promoter demethylation of *IRF4*, a previously unknown tumor suppressor gene in this context. In support of our findings, it is interesting to note that significantly higher methylation in *IRF4* was previously observed in HER2+ breast tumors in relation to normal breast tissues [61], and that high IRF4 expression associates with improved outcome in HER2+ node-negative BC [62]. While our work shows that *IRF4* silencing indeed promotes tumor growth, further studies will be needed to fully decipher the exact anti-tumor program driven by IRF4 in BC, as well as the signaling pathways controlling its expression in both normal and BC tissues.

Taken together, our study offers a new therapeutic avenue for BC treatment by simultaneously targeting the methionine cycle and DNA methylation via the combined actions of ERRα and DNMT inhibitors, while potentially reducing the toxic side-effects associated to high doses of demethylating agents. Finally, it will be interesting to establish whether this combination therapeutic approach could be beneficial for other non-hematopoietic cancers.

## Materials and methods

Details of all methods are found in Supplementary Information.

### Accession numbers

RRBS datasets from mouse tumor xenografts have been deposited in the NCBI Gene Expression Omnibus (GEO; https://ncbi.nlm.nih.gov/geo/) under the accession number GSE149603.

## Supplementary information

Supplementary Information

Supplementary Tables
