# Supplementary material for: Inhibition of DNMT1 and ERRα crosstalk suppresses breast cancer via derepression of IRF4
Source: Oncogene. 2020 Aug 27;39(41):6406–20. doi: 10.1038/s41388-020-01438-1 (PMC7544553; doi:10.1038/s41388-020-01438-1)

**Inhibition of DNMT1 and ERRα Crosstalk Suppresses Breast Cancer via Derepression of *IRF4***

**Mathieu Vernier, Shawn McGuirk, Catherine R. Dufour, Sydney Wan, Etienne Audet-Walsh, Julie St-Pierre, Vincent Giguère**

**Supplementary Information**

**Document S1. Materials and Methods, Supplementary References, Supplementary Figs. 1-7 and Tables 1-4 (one Excel file)**

**Materials and Methods**

**Cell lines and reagents**

BT474, SKBR3, MCF-7, MDA-MB-231, MDA-MB-436 and MDA-MB-468 cell lines were originally obtained from American Type Culture Collection (ATCC) and maintained in DMEM supplemented with 10% fetal bovine serum (FBS). ERRα WT and null MEFs derived from mice with a C57BL/6J genetic background were maintained in DMEM supplemented with 10% FBS. NIC-5231 and NIC-5257 cells were maintained in DMEM supplemented with 5% FBS, 5 ng ml^−1^ EGF, 1 μg ml^−1^ hydrocortisone, 5 μg ml^−1^ insulin (all from Wisent) and 35 μg ml^−1^ Bovine Pituitary Extract (Hammond Cell Tech). All cell lines were assessed for mycoplasma contamination after thawing as well as once per month. C29 and 5-aza-2’-deoxycytidine (Sigma Aldricht) treatments were carried out at a concentration of 5 μM each for 24 hours unless otherwise indicated. Bafilomycin A1 (Sigma Aldricht) treatment was carried out at a concentration of 20 nM. Cells treated with lapatinib (Sigma Aldricht) were carried out at 1 μM for 24 hours. Pools of siRNAs against ERRα, DNMT1 and control (ON-Target-Plus siRNA pool) were obtained from Dharmacon. Transfections were performed with Hiperfect (Qiagen). Short hairpin RNAs against *IRF4* were obtained from Sigma-Aldricht (shIRF4_1: TRCN0000014764; shIRF4_2: TRCN0000014765). pLPC-DNMT1 was kindly given by Dr Gerardo Ferbeyre (CRCHUM, Montréal, Québec). ERRα ChIP assays were performed using an anti-ERRα rabbit monoclonal antibody (Abcam, 2131-1) and control anti-rabbit IgG antibody (Santa Cruz, sc-2027). Western blots on total cellular extracts were performed using the following antibodies: ERRα (Abcam, ab76228); DNMT1 (Abcam, ab19905); DNMT3A (Abcam, ab23565); TET3 (Abcam, ab174862); MAT1A (Abcam, ab129176; MAT2A (Abcam, ab77471); LC3B (Cell Signaling, 2775S); and Tubulin (Millipore, #05-661).

**Cell proliferation assay**

2x10^3^ cells were seeded into 96-well plates. The next day, cells were treated with C29 (5 μM), 5-azadC (at the indicated concentrations) or a combination of both drugs and transferred to an IncuCyte ZOOM Live cell analysis system (Essen BioScience). Otherwise, cells were treated with lapatinib (0.25 μM) and/or 5-azadC (3 μM). Cell proliferation was monitored using IncuCyte ZOOM phase-contrast quantification software (2014B). For each assay, three independent experiments each with five replicates were performed and one representative experiment is shown. Data represent means ± SEM. ***p < 0.01,* ****p < 0.001*; Student’s t-test.

**Chromatin immunoprecipitation**

For ChIP analyses, chromatin was prepared from SKBR3 cells cultured for 24 hours with C29 (5 μM) prior to harvesting. Standard ChIP assays were performed as described previously [1] and quantification of ChIP enrichment by real-time quantitative PCR was carried out using the LightCycler480 instrument (Roche) with specific ChIP-qPCR primers listed in Supplementary Table 4. ChIP enrichments were normalized against background enrichment on anti-IgG antibody ChIP control and enrichment over a negative control unbound region. Results are displayed in a representative graph of three independent experiments performed in triplicates. Statistical significance of standard ChIP-qPCR data was performed using unpaired Student's *t*-tests.

**Quantitative RT-PCR**

RNA was extracted and purified using the RNeasy Mini Kit (Qiagen, #74106) following the manufacturer’s protocol. Reverse transcriptase reactions were performed using SuperScript II (Invitrogen). Samples were then analyzed using SYBR-green-based qRT-PCR on a LightCycler 480 instrument (Roche) with specific primers listed in Supplementary Table 4. Relative gene expression levels were normalized against the expression of *TBP*. Results reflect three independent experiments performed in triplicate. Data represent means ± SEM. **p < 0.05,* ***p < 0.01*; Student’s t-test.

**Metabolomics**

Steady-state metabolite abundances were determined using an LC/MS system with unlabeled media. Metabolite abundances were determined by washing 70-80% confluent 10-cm plates of cells with 150 mM ammonium formate at 4°C, quenched in 80% HPLC-grade methanol at -20°C on dry ice, after which the cell slurry was quickly transferred to tubes equilibrated in liquid nitrogen. Then, the samples were phase-separated using acetonitrile, water, and dichloromethane after vigorous bead-beating and vortexing. The aqueous phase was collected and dried in a cold trap at -1°C. Once dry, pellets were maintained at -80°C and solubilized in HPLC water immediately before injection into an Agilent 6430 Triple Quadrupole LC/MS system coupled to ultra-high-pressure liquid chromatography (UHPLC, 1290 Infinity LC System) separation for fast targeted analysis. Data were analyzed using MassHunter software (Agilent). Results reflect three independent experiments each performed with five replicates. Data represent means ± SEM. **p < 0.05,* ***p < 0.01*, ****p < 0.001*; Student’s t-test.

For stable isotope tracer analyses (SITA), cells were seeded in 6-well dishes to achieve 70-80% confluency after 48 hours. Media was then replaced by DMEM without methionine, serine and glycine (Wisent, custom-made, #319-109-CL) supplemented with 10% FBS, 25 mM glucose, 1X sodium pyruvate, 4 mM glutamine, and 0.2 mM ^13^C_5_^15^N-methionine to equilibrate metabolism for 2 hours (metabolite profiling) or 24 hours (DNA methylation profiling). C29 was present in the media throughout. Metabolites were then purified and processed as described above for steady state metabolites. Results reflect three independent experiments each performed with five replicates. Data represent means ± SEM. **p < 0.05,* ***p < 0.01*, ****p < 0.001*; Student’s t-test.

**DNA methylation quantification**

2x10^5^ cells were seeded into 6-cm plates. The next day, cells were treated with C29 (5 μM), 5-azadC (at the indicated concentrations) or a combination of both for 24 hours and genomic DNA was purified using a DNeasy Blood and Tissue purification kit (Qiagen, #69506) following the manufacturer’s protocol. For colorimetric assays, 5-methylcytosine was detected using the MethylFlash Methylated DNA 5-mC Quantification Kit (Epigentek, #P-1034) following the manufacturer’s protocol. Results reflect three independent experiments performed in triplicate.

For DNA methylation profiling in studies with labeled methionine, 5-methylcytosine quantification involved exposure of extracted DNA to acid hydrolysis following a technique developed previously [2]. Briefly, 1 μg DNA was dried at 40°C under nitrogen gas. 100 μL of formic acid was added to the dry pellets and incubated at 130°C for 3.5 hours. After cooling, the acid was dried off at 40°C under nitrogen gas. Dry pellets were resuspended in HPLC water at room temperature for 20 minutes before injection into an Agilent 6430 Triple Quadrupole LC/MS system coupled to ultra-high-pressure liquid chromatography (UHPLC, 1290 Infinity LC System) separation for fast targeted analysis. Data were analyzed using MassHunter software (Agilent). Results reflect three independent experiments each performed with five replicates. Data represent means ± SEM. **p < 0.05,* ***p < 0.01*, ****p < 0.001*; Student’s t-test.

**Reduced Representation Bisulfite Sequencing (RRBS)**

Genomic DNA was extracted from tumor xenografts and purified using the DNeasy Blood and Tissue purification kit (Qiagen, #69506) following the manufacturer’s protocol. For each condition, 2 replicates consisting of a pool of DNA from 2 mice were used. RRBS library preparation and deep sequencing were performed by Diagenode (Denville, New Jersey). Sequencing was performed on an Illumina HiSeq 3000/4000 with 40 million pre-filtered reads per lane, covering 3-4 million CpGs on average. Reads were aligned to the murine reference genome mm10/GRCm38 using bismark v0.20.0. Methylkit v1.7.0, a R/Bioconductor package, was used to perform the differential methylation analysis between the mouse treatment groups. Motif analysis was performed in-house using the command findmotif_genome within the Homer software package [3] whereby the promoters having DMRs for each condition were used as input.

**Methylation-specific quantitative PCR (MS-qPCR)**

Promoter methylation of the *IRF4* gene was investigated using quantitative methylation-specific PCR (Q-MSP) as previously described [4]. Briefly, cells were treated with C29 (5 mM), 5-azadC

(3 mM) or a combination of both drugs for 7 days prior to genomic DNA extraction using the DNeasy Blood and Tissue purification kit (Qiagen, #69506) following the manufacturer’s protocol. Then, genomic DNA was converted by bisulfite treatment using the EpiTect bisulfite kit (Qiagen, #59104) following the manufacturer’s protocol. Hypermethylation of IRF4 CpG islands was then examined by real-time PCR amplification of bisulfite-modified DNA using oligonucleotide primers designed to target either methylated or unmethylated DNA (primers are listed in Supplementary Table 4). *IRF4* CpG islands and methylation-specific qPCR primers were designed with the online software MethPrimer (https://www.urogene.org/methprimer/). The performance of the Q-MSP primers was evaluated by running a standard curve and melting curve before they were applied for quantitative gene methylation analysis. Relative DNA quantity was normalized using primers complementary to a genomic region of the β-actin-encoding gene, Actb, that does not contain CpG dinucleotides but does contain non-CpG cytosines. Results reflect three independent experiments performed in triplicate. Data show means ± SEM. **p < 0.01, ***p < 0.001; Student’s t-test.

**Xenografts**

To determine the ideal concentration of 5-azadC to be used, we first performed a pilot study where 1, 2.5, 5 or 10 mg/kg of 5-azadC were injected intraperitoneally every two days in NSG mice 5-7 weeks old. The maximum tolerable dose (MTD) was defined as the dose at which all animals survived for 4 weeks, and at which overt clinical signs of toxicity (loss of body weight, general weakness, severe hair loss, or shaggy coat) were absent. While treatments with 5 or 10 mg/kg of 5-azadC were lethal for most of the mice tested, a dosage of 2.5 mg/kg induced loss of body weight and signs of weakness. Treatment with 1 mg/kg every 2 days was well tolerated and was taken as the MTD. Then, 1x10^6^ NIC-5231 cells were injected into the mammary fat pad of 5-7-week-old NSG mice (n=20 mice total). When tumors were palpable, mice were randomly assigned to either a control group (vehicle) or a treatment group (n=5 mice per group; the investigator was not blinded during group allocation). C29 (10 mg/kg) and/or 5-azadC (1 mg/kg) were resuspended in saline/30%PEG/5%DMSO and administered by intraperitoneal injection every 2 days. Tumor size was assessed in a blinded fashion using calliper measurements and the experiment was terminated when the largest tumor had reached the maximum tumor size limit (1.5 mm^3^). All animal studies were approved by the McGill University Animal Care Committee (UACC).

**Biostatistical analysis**

Biostatistical analysis was conducted on three independent cohorts of breast cancer patients comprising baseline gene expression profiles of primary breast tumors (GSE2034; E-TABM-158; GSE24450). Unsupervised hierarchical clustering of tumor samples was performed with Gene Cluster 3.0 using the hierarchical linkage method after filtering data to keep expression of the genes of a previously published ERRα gene signature [5] (Supplementary Table 1) and visualized using JavaTreeView [6]. Vertical scatter plots were generated with GraphPad Prism 8 by comparing relative gene expression of the indicated genes between the two clusters of patients identified in each cohort.

**Supplementary References**

1 Chaveroux C, Eichner LJ, Dufour CR, Shatnawi A, Khoutorsky A, Bourque G *et al*. Molecular and genetic crosstalks between mTOR and ERRα are key determinants of rapamycin-induced non-alcoholic fatty liver. *Cell Metab* 2013; 17: 586-598.

2 Maddocks OD, Labuschagne CF, Adams PD, Vousden KH. Serine metabolism supports the methionine cycle and DNA/RNA methylation through de novo ATP synthesis in cancer cells. *Mol Cell* 2016; 61: 210-221.

3 Heinz S, Benner C, Spann N, Bertolino E, Lin YC, Laslo P *et al*. Simple combinations of lineage-determining transcription factors prime cis-regulatory elements required for macrophage and B cell identities. *Mol Cell* 2010; 38: 576-589.

4 Nikolaidis G, Raji OY, Markopoulou S, Gosney JR, Bryan J, Warburton C *et al*. DNA methylation biomarkers offer improved diagnostic efficiency in lung cancer. *Cancer Res* 2012; 72: 5692-5701.

5 Chang CY, Kazmin D, Jasper JS, Kunder R, Zuercher WJ, McDonnell DP. The metabolic regulator ERRα, a downstream target of HER2/IGF-1R, as a therapeutic target in breast cancer. *Cancer Cell* 2011; 20: 500-510.

6 Saldanha AJ. Java Treeview--extensible visualization of microarray data. *Bioinformatics* 2004; 20: 3246-3248.

**Supplementary Fig. Legends**

**Supplementary Fig. 1. ERRα targets genes involved in DNA methylation and the methionine cycle. a.** Duplicate ChIP-seq binding profiles of ERRα on the indicated genes in both BT474 and SKBR3 cells. The location of primers used for standard ChIP-qPCR validation of ERRα binding to these genes in Figure 1b are shown. **b.** Immunoblots of ERRα and DNMT1 in the ER+ BC cell line MCF7 post-transfection for 48 hours with siRNAs against ERRα. Tubulin is shown as a loading control. **c-e.** Immunoblots of ERRα and DNMT1 in the TNBC cell lines MDA-MB-231 (**c**), MDA-MB-436 (**d**) and MDA-MB-468 (**e**) post-transfection for 48 hours with siRNAs against ERRα. Tubulin is shown as a loading control.

**Supplementary Fig. 2. ERRα is a driver of DNA methylation.** Quantification of 5-methylcytosine by colorimetric assays in ERRα KO MEFs compared to WT. Results reflect three independent experiments performed in triplicate. Data represent means ± SEM. **p < 0.05*; Student’s t-test.

**Supplementary Fig. 3. Correlation between ERRα activity and genes linked to DNA methylation and the methionine cycle.** Relative expression of key genes involved in the methionine cycle and DNA methylation between patients with low (blue) and high (yellow) ERRα activity for each BC patient cohort. Gene expression values were extracted from microarray data after normalization and were log2 transformed. Data show means ± SEM; Student’s t-test.

**Supplementary Fig. 4. Dual inhibition of ERRα and DNMT in ER+ and TNBC cell lines augments BC cell growth hindrance *in vitro.* a-d.** Immunoblots of ERRα and DNMT1 in the ER+ BC cell line MCF-7 (**a**) and in the TNBC cell lines MDA-MB-231 (**b**), MDA-MB-436 (**c**) and MDA-MB-468 (**d**) after treatment with C29 (5μM) and/or 5-azadC (3μM) for 24h. **e** Immunoblots of ERRα, DNMT1 and DNMT3a in SKBR3 cells after treatment with lapatinib (1uM) and 5-azadC (3μM) for 24h. **f.** Normalized cell index curves representing proliferation of SKBR3 cells in the presence of lapatinib (0.25 uM) and/or 5-azadC (3μM). Three independent experiments each with five replicates were performed and one representative experiment is shown. Data shown in **f** represent means ± SEM.

**Supplementary Fig. 5. RRBS analysis in a mouse NIC-5231 CDX model treated with ERRα and/or DNMT inhibitors. a-c** Percentage of hyper and hypomethylated DMCs in tumors extracted from mice treated with either C29 (**a**), 5-azadC (**b**), or both drugs (**c**) for 20 days. **d-f** Percentage of DMCs found in introns, promoters, exons or intergenic regions in tumors extracted from mice treated with either C29 (**d**), 5-azadC (**e**), or both drugs (**f**) for 20 days. **g-i** KEGG pathway enrichment analysis of the total list of genes with DMCs, regardless of the genomic location of the DMCs relative to genes, in tumors extracted from mice treated with either C29 (**g**), 5-azadC (**h**), or both drugs (**i**) for 20 days.

**Supplementary Fig. 6. *IRF4* methylation status in cancer. a** Box plot showing the pan-cancer methylation status of *IRF4* from the TCGA project using a mean aggregation of all 16 CpGs referenced within the SMARTapp. The outer limits of the box represent the 25^th^ (lower quartile) and 75^th^ percentile (upper quartile) with the median value shown inside. Data points outside the limits of the whiskers are outliers. *p < 0.05, **p < 0.01, ***p < 0.001, ****p < 0.0001, ns=not significant; Wilcoxon rank sum test and Benjamini-Hochberg adjusted. **b** Kaplan-Meier survival curves derived from the SMARTapp illustrating the correlation between the methylation status (M-value) of the indicated *IRF4*-associated CpG sites with BC patient overall survival. **c** Spearman correlation curves obtained from the SMARTapp showing the association between the methylation status (M-value) of the indicated *IRF4*-associated CpG sites and *IRF4* gene-level expression in BC patients (n=853).

**Supplementary Fig. 7. Knockdown of the silenced tumor suppressor gene IRF4 has no effect on BC cell growth.** Normalized cell index curves representing proliferation of SKBR3 cells infected with either a control shRNA (shNTC) or 2 different shRNAs against IRF4 in the presence of vehicle (DMSO). The *IRF4* gene is already hypermethylated and repressed in BC cells and in a control experiment we show that shRNA-mediated silencing of IRF4 has no further effect. Data represents means ± SEM of one experiment with five replicates performed.

**Supplementary Table 1.** A gene signature reflective of ERRα activity used to separate patients with low versus high ERRα activity in BC patients.

**Supplementary Table 2.** Total list of genes with promoter DMRs found modulated in tumors from an established mouse CDX model treated with either C29, 5-azadC or a combination of both drugs compared to vehicle (DMSO).

**Supplementary Table 3.** List of the 890 genes with promoter DMRs found uniquely modulated in tumors of an established mouse CDX model co-treated with C29 and 5-azadC. Within this list, 9 of 51 TF's were found to associate with poor outcome in BC patients determined using the SMARTapp of which 6 were found hypomethylated in response to C29 and 5-azadC co-administration.

**Supplementary Table 4.** Human primers used for ChIP-qPCR, qRT-PCR and MS-qPCR analysis.


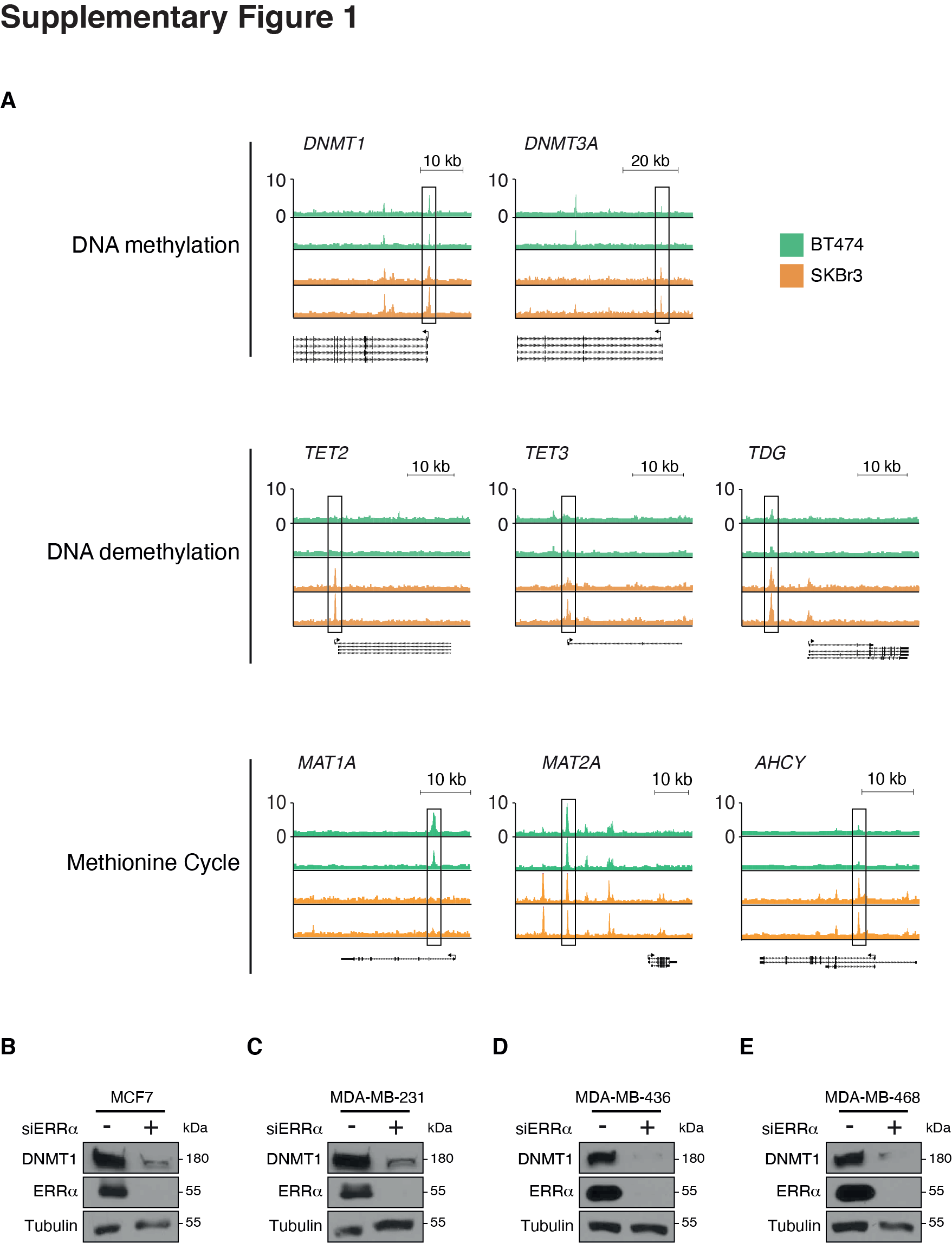


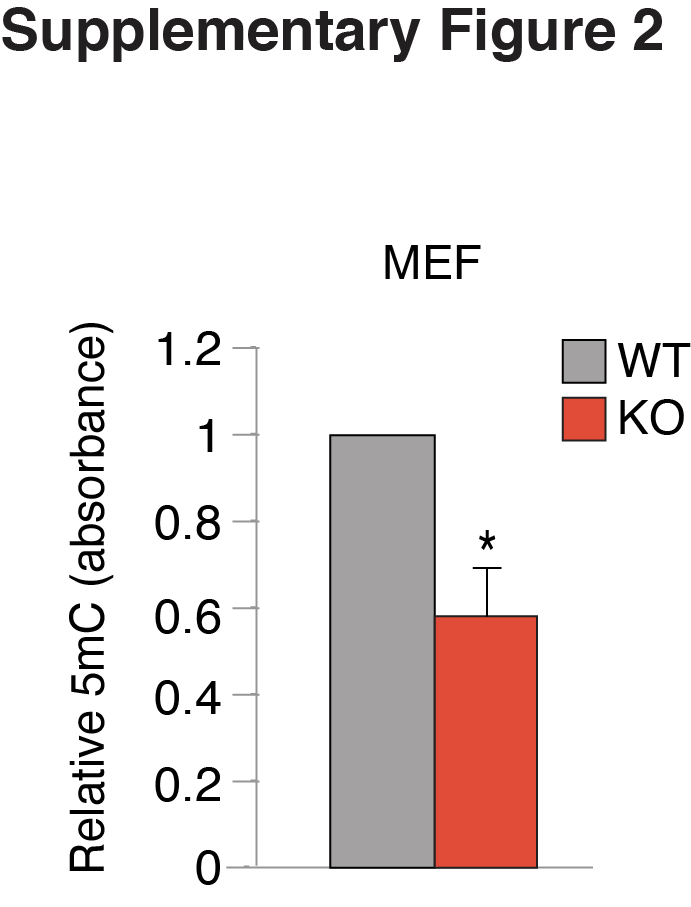


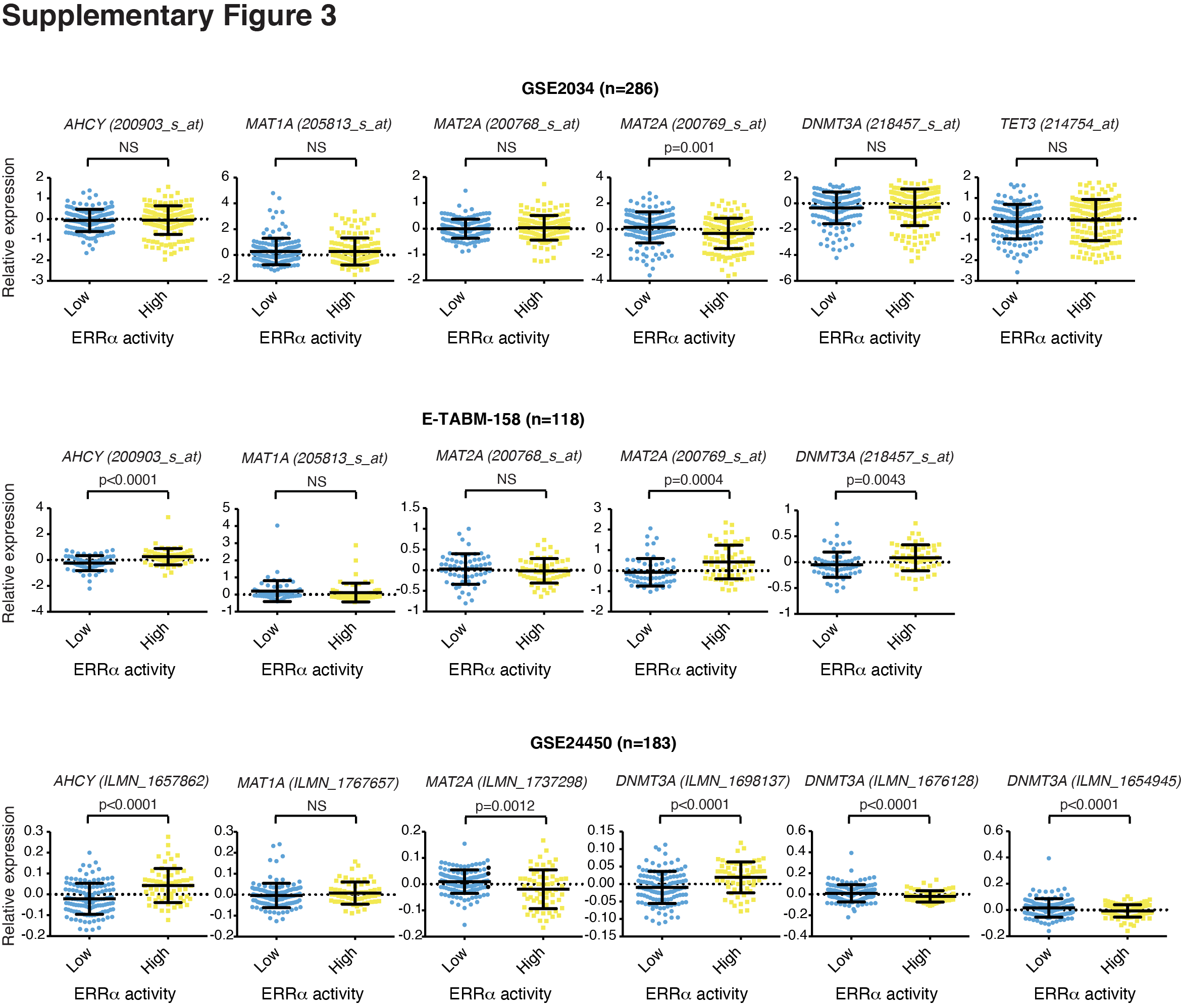


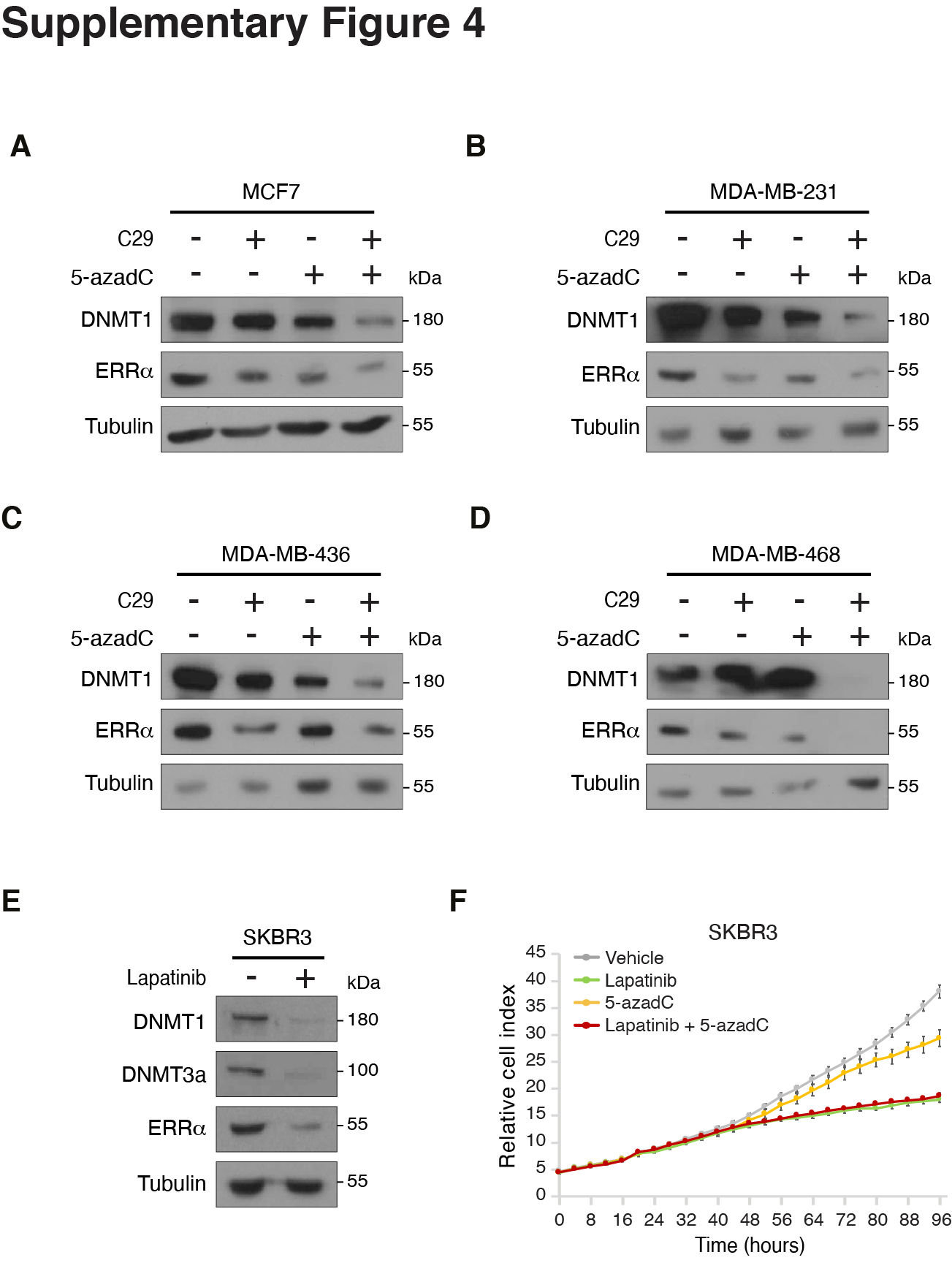


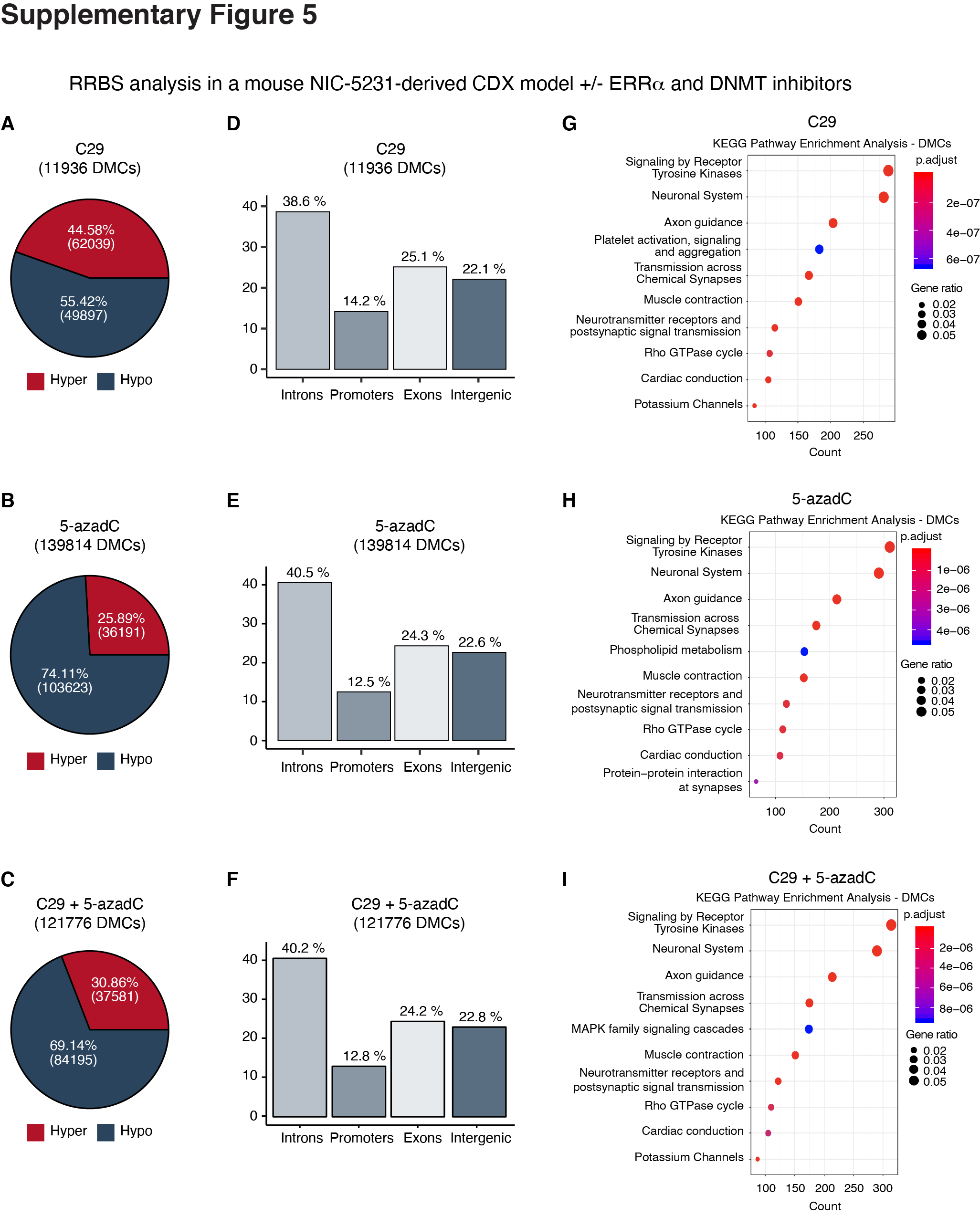


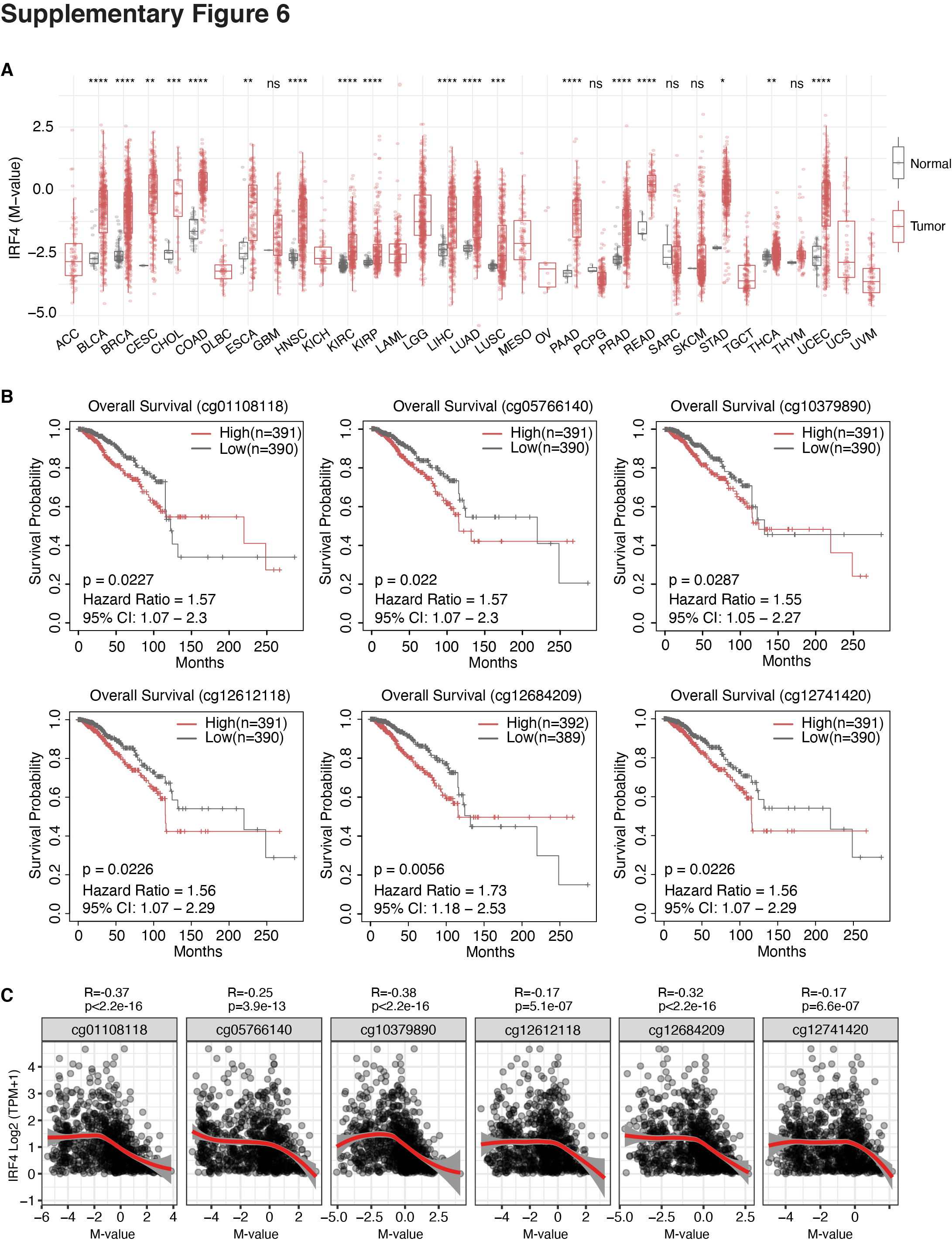


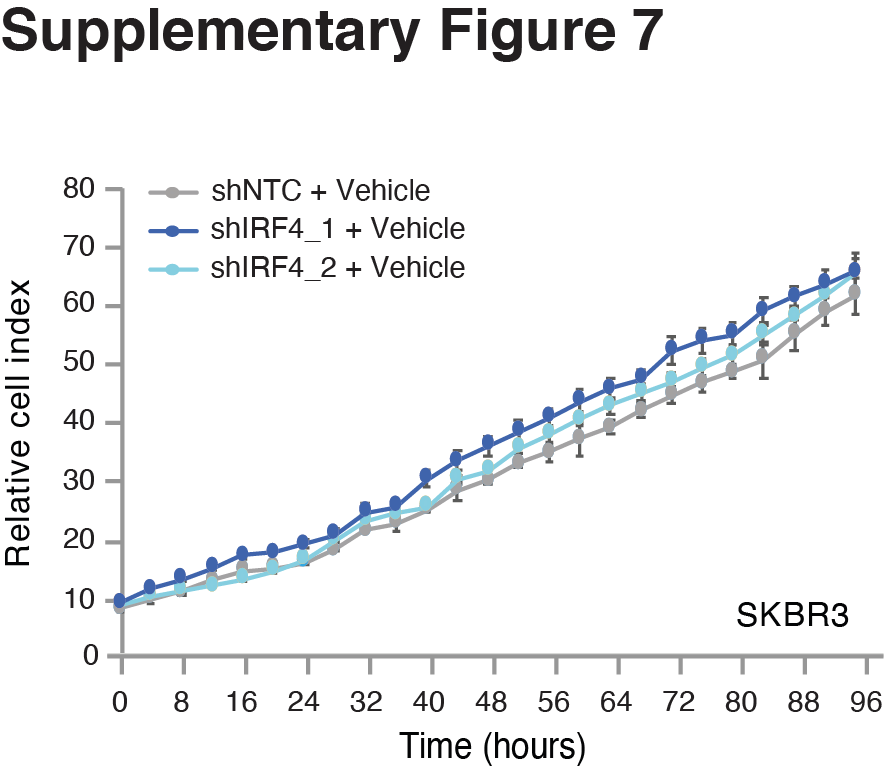

Supplement: Supplementary file 1 — Supplementary Information [file 41388_2020_1438_MOESM1_ESM.docx]
